# Dimensional and hierarchical models of depression using the Beck Depression Inventory-II in an Arab college student sample

**DOI:** 10.1186/1471-244X-10-60

**Published:** 2010-07-29

**Authors:** Fawziyah A Al-Turkait, Jude U Ohaeri

**Affiliations:** 1 Department of Psychology, College of Education, Public Authority for Applied Education and Training, Kuwait, P.O. Box 117, Safat, 13002, Kuwait; 2 Department of Psychiatry, Psychological Medicine Hospital, Gamal Abdul Naser Road, P.O. Box 4081, Safat, 13041, Kuwait

## Abstract

**Background:**

An understanding of depressive symptomatology from the perspective of confirmatory factor analysis (CFA) could facilitate valid and interpretable comparisons across cultures. The objectives of the study were: (i) using the responses of a sample of Arab college students to the Beck Depression Inventory (BDI-II) in CFA, to compare the "goodness of fit" indices of the original dimensional three-and two-factor first-order models, and their modifications, with the corresponding hierarchical models (i.e., higher - order and bifactor models); (ii) to assess the psychometric characteristics of the BDI-II, including convergent/discriminant validity with the Hopkins Symptom Checklist (HSCL-25).

**Method:**

Participants (N = 624) were Kuwaiti national college students, who completed the questionnaires in class. CFA was done by AMOS, version 16. Eleven models were compared using eight "fit" indices.

**Results:**

In CFA, all the models met most "fit" criteria. While the higher-order model did not provide improved fit over the dimensional first - order factor models, the bifactor model (BFM) had the best fit indices (CMNI/DF = 1.73; GFI = 0.96; RMSEA = 0.034). All regression weights of the dimensional models were significantly different from zero (P < 0.001). Standardized regression weights were mostly 0.27-0.60, and all covariance paths were significantly different from zero. The regression weights of the BFM showed that the variance related to the specific factors was mostly accounted for by the general depression factor, indicating that the general depression score is an adequate representation of severity. The BDI-II had adequate internal consistency and convergent/discriminant validity. The mean BDI score (15.5, SD = 8.5) was significantly higher than those of students from other countries (P < 0.001).

**Conclusion:**

The broadly adequate fit of the various models indicates that they have some merit and implies that the relationship between the domains of depression probably contains hierarchical and dimensional elements. The bifactor model is emerging as the best way to account for the clinical heterogeneity of depression. The psychometric characteristics of the BDI-II lend support to our CFA results.

## Background

Findings of the multi-domain nature of depressive symptomatology have led to a search for new descriptive and explanatory models in the attempt to identify parsimonious and distinct dimensions of depression, while maintaining the breadth necessary to encompass the full range of features observed clinically [1,2]. These studies involve the techniques of exploratory factor analysis (EFA) and confirmatory factor analysis (CFA). An understanding of the dimensions of depressive symptoms could facilitate valid and interpretable comparisons across cultures [3]. In addition, specific domains of depression have been linked with genetic vulnerability [4], dexamethasone non-suppression [5], localization of brain lesions [6], clinical outcome in physical illnesses [7], and characterization of subjects with suicidal and behavior disorders [8,9].

**Figure 1:**
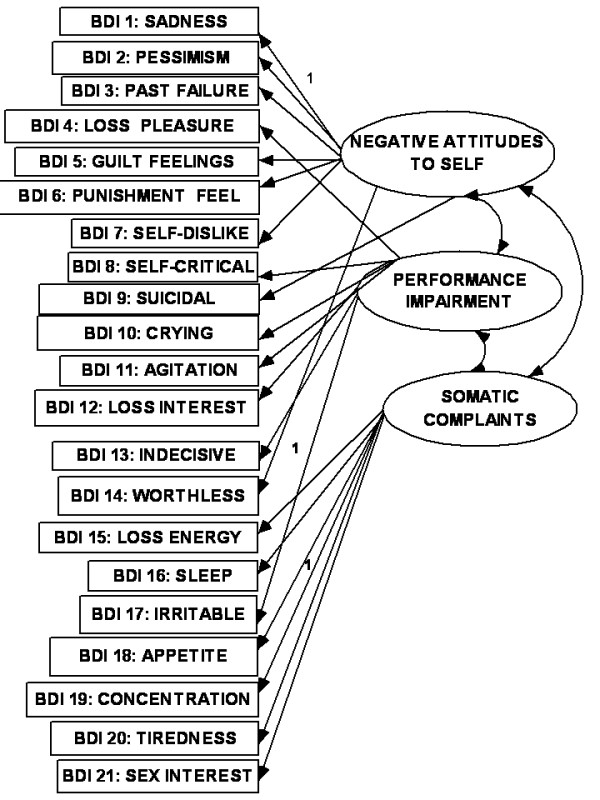
**3-factor lower order model**.

**Figure 2:**
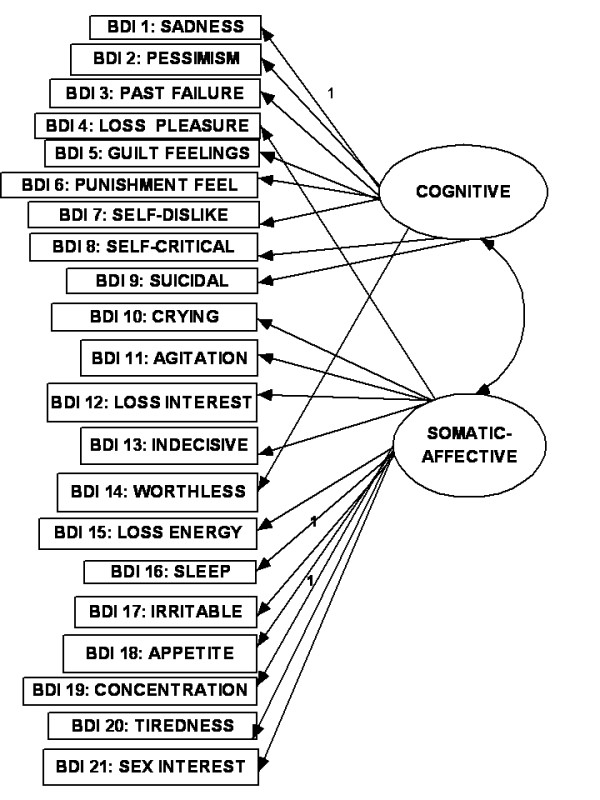
**Somatic-affective/cognitive model**.

**Figure 3:**
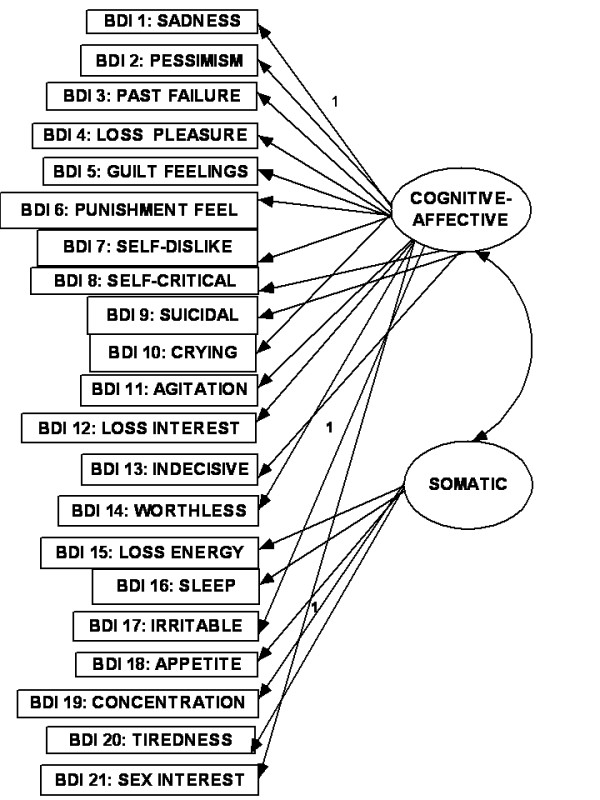
**Cognitive-affective/somatic model**.

As the most frequently used self-rating scale in depression [10], the Beck Depression Inventory (BDI) has received the greatest attention in these reports [1]. The original BDI has been revised to correspond with the DSM-IV criteria [11] for depression (BDI -II: Beck et al [12]). In a meta-analysis of factor structures of the original version of the BDI, Shafer [1] found that the average number of factors extracted was four (range 2-7) and average range of variance explained was 46%. About 30% of studies were student samples. The three most consistent domains of depression were, "negative attitudes to self", "performance impairment" and "somatic complaints". In CFA studies using the BDI -II, the dimensional model with these three first-order factors have been shown to have adequate fit to the data [13,14] (see Fig 1). The BDI-II was originally validated using an outpatient sample (N = 500) and an undergraduate sample (N = 120)[12]. Each sample yielded two factors in EFA, using items that loaded ± 0.35 on the corresponding factors. The factors for the outpatient sample were labeled "somatic -affective" (SA) and "cognitive" (C) (i.e., SA-C model). The factors for the undergraduate sample were labeled "cognitive-affective" (CA) and "somatic" (S) (i.e., CA-S model). In subsequent CFA studies using all the items of the BDI-II, these two-factor models were confirmed for a clinically depressed outpatient group [15] (see Fig 2) and for samples of undergraduate students [16,17] (see Fig 3). However, in a large sample of Canadian students [18], the two-factor solution was rather similar to that from Beck's outpatient sample (BDI-II items 1-3, 5-9 and 13-14 loaded on the "C-A" factor; while items 4,10-12 and 15-21 loaded on the somatic-vegetative factor). Although several studies have supported these two-factor solutions in FA using clinical populations [19-25] and student populations [26-29], some reports were not supportive [30-35]. In other words, the factorial validity of the BDI-II is still controversial [32,35], and there is no formal assignment of items to scales [1]. This controversy is evident in the few reports on the factor analysis of the BDI-II from the Middle East. While one Iranian report on students supported the two-factor model [27], another Iranian study reported a five-factor solution [35]. One study from the Arabian Gulf state of Bahrain [36] (with similar Arabic language dialect as Kuwait) found three factors ("cognitive-affective", "overt emotional upset", and "somatic -vegetative") which were much similar to the original three factors (except that the Bahraini BDI-II items: 4,8,10-13,17 constituted the "overt emotional upset" domain).

**Figure 4:**
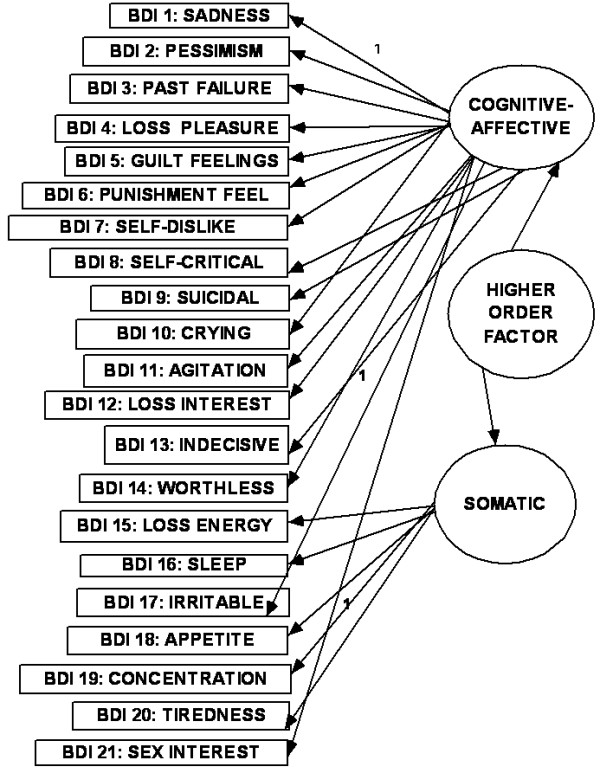
**Higher order model**.

**Figure 5:**
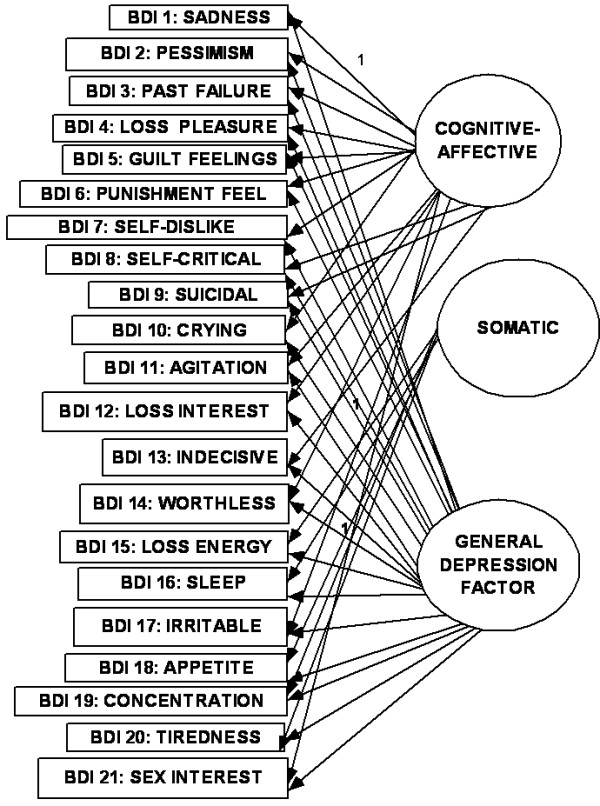
**Bifactor model using CA-S model**.

The relationship between the items of any questionnaire where there are diverse indicators of a complex construct can be described as existing in dimensional and hierarchical models [1,14,37]. In the dimensional model, the first-(or lower-) order factors (or domains) exist on only one plane in which they may freely relate with one another. In the hierarchal model, the factors are disposed in two or more levels (or hierarchs) in which the relationship between the lower order factors is restricted (i.e., either no relationship or indirect relationship through a higher-order factor). There seems to be an emerging consensus in the CFA literature on the BDI that, while the classical first-order multi-factor models (i.e. dimensional models) (e.g., Figs 1, 2 and 3) provide adequate fit to the data, the hierarchical models tend to provide better fit indices [13-15,38-43]. It has been suggested that the first-order dimensional models are probably too limited to fully describe the heterogeneity observed among people with depression [2]. Of the two hierarchical models described for depression, the higher-order model has received more attention in the literature than the bifactor model[14]. In the higher order model [44], the lower order factors/sub-factors (e.g., "C-A" and "S") are modeled as differential elements (or facets) of a general depression (second - order) factor that permeates the instrument as a whole; but this general factor is not directly related to the individual (observed) items of the BDI-II (see Fig 4). The bifactor approach assumes a general factor underlying all variables (e.g., all items of the BDI-II); but in addition it includes a number of uncorrelated group factors consisting of two or more variables (e.g., "C-A" and "S") (see Fig 5). The bifactor approach was initially developed in the context of research on cognitive abilities by Holzinger and Swineford [45], but has been extended to psychopathology by workers in the field of externalizing disorders [44], depression [46] and health-related quality of life [47]. In these hierarchical models, the lower order factors reflect the specific contents of the mood state, and provide a basis for differentiation between patient groups, while the upper level reflects their common characteristics [48,49].

There is a paucity of studies that have used the bifactor approach to compare the various first-order factor models of the BDI-II [14]. Since over 30% of factor analytic studies of the BDI were based on samples of college students [1], we have studied an undergraduate sample in order to make our findings comparable with the international literature. Several authors have expressed the need to use the BDI-II to test the models in student populations across cultures because of their homogeneity and comparability [14,16-18,26,27,29]; and the sample of college students was found to be useful in the original validation studies of the BDI-II because it is a close approximation to the general population [12]. Also, our use of symptom-level data has the potential to expose greater variation in the data than disorder-level variables [2].

The objectives of the study were: (i) using the responses of a sample of Arab college students to the Beck Depression Inventory (BDI-II) in CFA, to compare the "goodness of fit" indices of the original dimensional three-and two-factor first-order models, and their modifications (Figs 1, 2 and 3), with the corresponding hierarchical models (i.e., higher - order and bifactor models) (Figs 4 and 5). We also examined the Bahraini model [36] because it is the only one from our region, and the Dozois model from college students [18], because it was similar to the original two-factor model from an outpatient sample; (ii) to assess the following psychometric characteristics of the BDI-II, in comparison with the international data: internal consistency, item mean scores, corrected item-total correlations, and convergent/discriminant validity with the anxiety and depression subscale scores of the Hopkins Symptom Checklist (HSCL-25) [50].

Based on the literature [14,17,35,42,43,46], we hypothesized that the hierarchical models would have better fit indices than the dimensional first-order models; the bifactor models would have the best fit indices; and the psychometric characteristics of the BDI-II would be adequate.

## Method

### Setting, subjects and procedure

Kuwait is a conservative Arab country situated in the Arabian Gulf. Study participants were students of the College of Education, Public Authority for Applied Education and Training (PAAET), Kuwait. This is a four-year program degree - awarding institution with a total population of 8000 students (2000 men, 6000 women).

Following the example of several studies with similar objectives in the literature [12-19] (some of which recruited participants by newspaper advertisements), our methodology did not require a probability sample, especially as this was not a study of the prevalence of the disorder.

The 624 participants consisted of 182 (29.2%) men and 442 (70.8%) women from all the years of study. This was fairly similar to the ratio of men to women in the entire student population. They were aged 18 to 38 years (mean = 20.8; SD = 2.9; mode and median = 20 years).

Participants completed the questionnaires in the 2007/2008 academic session. They were approached in class at the end of lectures by the research team. In order to include students in all the disciplines, the classes chosen were compulsory general studies' courses. One general studies' course was chosen per year of study for the four years of study. They self-completed the questionnaires anonymously. First, the objectives of the study were explained. The students were duly informed that they were free to decline to participate, and that there would be no penalty for refusing to participate. They gave verbal informed consent. The study was approved by the institutional review panel of the PAAET.

### Beck Depression Inventory (BDI -II)

Like the original BDI, the BDI-II has 21 items, each of which consists of four self-evaluative statements in a time frame of two weeks, and scored 0 to 3, with increasing scores indicating greater depression severity. Responses are summed to yield a total score that ranges from 0 to 63. The BDI-II has been used in previous studies of samples of students and primary health care attendees in the Arabian Gulf, including Kuwait [36,51,52], and an Arabic translation exists, produced by the method of back-translation. The internal consistency (Cronbach's alpha) for the 21 items, using the responses of all participants was 0.83.

### Hopkins Symptoms Checklist-25 [50]

The HSCL-25 is presented in the context of convergent/discriminant validity for our primary analyses on psychometric characteristics. The first ten items of the questionnaire concern anxiety while the remaining 15 items relate to depression. The response options for each item are: "not at all", "a little", "quite a bit", and "extremely", rated 1-4 respectively. Higher scores indicate worse mental functioning. Three summed scores are calculated: the total score is the average of all 25 items; the anxiety score is the average of the 10 anxiety items; while the depression score is the average of the 15 depression items. The internal consistency (Cronbach's alpha values) of the questionnaire for the responses of all 624 participants are as follows: (i) for the 25 items, 0.91; (ii) for the 10 anxiety items, 0.85; and (iii) for the 15 depression items, 0.86.

### Data analysis

Data were analyzed by the Statistical Package for Social Sciences, version 15 (SPSS Inc., Chicago, Illinois). Structural equation modeling (SEM) operations (CFA) were done by Analysis of Moments Structures (AMOS), version 16 [53].

The CFA operations involved comparison of "fit" indices of BDI-II models from the previous studies earlier highlighted. These were: (i) the first - (or lower-) order three-factor model (Fig 1); (ii) the two-factor "SA-C" model (Fig 2); (iii) the two-factor "CA-S" model (Fig 3); (iv) the two-factor Dozois et al model [18]; (v) the three-factor Bahrain model [36]; (vi) the higher order models of each of these lower - order factor models (Fig 4); (vii) the bifactor model of each of the lower-order factor models (Fig 5); and (viii) the one-factor general depression model [35].

CFA is done by comparing the "goodness - of - fit" (GOF) indices of the various models. We used the maximum likelihood method of estimation (MLE). There are three broad types of GOF measures. Hooper et al [54] have suggested that, while there are no golden rules for assessment of model fit, reporting a variety of indices is necessary because different indices reflect different aspects of a model fit. In addition, fit indices may not perform uniformly across conditions [37]. Hence, in order to examine the robustness of our results and make our findings comparable with the international data, we chose fit indices from each of the three GOF measures [54], viz:

(a) Absolute fit indices, which do not make any comparison to a specified null model, or adjust for the number of parameters in the estimated model. From this group we chose the following: (i) the normed chi-square (chi-square or CMIN/DF). A value of <5 is considered adequate fit, while ≤2 is considered excellent fit [54]; (ii) GOF Index (GFI); (iii) adjusted GFI (AGFI). A value > 0.90 is considered adequate fit, while ≥0.95 is considered excellent fit, especially for small sample sizes [54]; (iv) Root mean square error of approximation (RMSEA). The recommended value is < 0.08 for adequate fit and < 0.06 for excellent fit [54];

(b) Incremental fit indices, which assess how well the estimated model fits relative to some alternative (null) model. From this group we chose: (v) Tucker-Lewis Index (TLI) or non-normed fit index (NNFI); and (vi) comparative fit index (CFI). The recommended value is > 0.90 for adequate fit and ≥0.95 for excellent fit; (c) Parsimony fit indices, which attempt to correct any overfitting of the model and evaluate the parsimony of the model compared to the GOF. From this group we chose: (vii) the parsimony comparative fit index (PCFI). The recommended value is > 0.6. Finally, we used (viii) the Akaike Information Criterion (AIC), a parsimony fit index, to make an overall comparison. A model with the smaller AIC has the better fit [54].

Assessment of multivariate normality of distribution of data in AMOS, using recommendations for item skewness (± 3) and kurtosis (± 7) [55] indicated that the data did not significantly deviate from normality. (For our sample, skew was 0.43-2.39; and kurtosis was - 0.28-6.87, all of which were within the recommended ranges).

Corrected total item correlations, measured by Pearson's correlation, were assessed after controlling for item overlap. Since the summary scores of the BDI factors and the anxiety/depression scores of the HSCL-25 were fairly normally distributed, gender differences in the BDI summary scores were assessed by t-tests, while their correlations with the HSCL-25 was done by Pearson's correlation. Comparison of our BDI mean scores with those of student data from other countries was done by effect size calculations. The level of statistical significance was set at P < 0.05.

## Results

The highlights of the CFA results are as follows (Table 1): (i) all the models met most of the criteria for good "fit", with CMIN/DF < 2.4, GFI > 0.90, AGFI > 0.90, PCFI > 0.74, and RMSEA < 0.05; (ii) for the dimensional first - order factor models, all regression weights (0.57-2.2) were significantly different from zero at 0.001 to 0.004 levels, two-tailed; and all covariance paths between the factors were significant. The standardized regression weights were 0.27 -0.60 for 20 items, and 0.14-0.16 for the item on concentration (BDI item 19). Further details for the standardized regression weights are as follows, using the results for Fig 1: 0.15-0.29 (for two items), 0.30-0.39 (three items), 0.40-0.49 (for eight items), 0.50-0.59 (five items) and 0.60 (for two items); (iii) the higher - order models and the one-factor model had identical fit indices; (iv) judging by the AIC values, the higher - order models did not result in better "fit" to the data (514.13), in comparison with the first - order factor models (481.7-510.4), especially as they had similar NNFI and CFI indices (each < 0.90 for the higher order models); (v) the bifactor versions (especially of the two-factor first order models) had the best fit indices, including the lowest AIC values. The bifactor version of the CA-S model (i.e., Beck et al [12] model from students' sample) had the best fit indices, with the lowest CMIN/DF and AIC values; (vi) the regression weights of the general factor of the bifactor models (0.51-2.5) were all significantly different from zero, mostly at 0.001 level, two-tailed. The standardized regression weights of the general factor for BDI items 1-18 were 0.35 -0.59 (i.e., accounted for 12.3% -35% of variance explained), 0.27 for BDI-II items 20 and 21(i.e., 7.3% variance) and 0.11 for item 19 (i.e., 1.2% of variance); (vii) the regression weights of the uncorrelated first-order factors of the bifactor models were not significantly different from zero. This suggests that the variance related to these specific factors was mostly explained by the general factor [47]. There was no particular tendency for cognitive symptoms to load higher than the somatic symptoms.

**Table 1:** Confirmatory factor analyses of the BDI-II: comparison of models by MLE method. N = 624

Models	CMNI/DF^1^	GFI^2^	AGFI^3^	TLI: NNFI^4^	CFI^5^	PCFI^6^	RMSEA^7^	AIC^8^	Regression weights: P values	Standardized regression weights
3-factor: Fig 1	2.11	0.94	0.93	0.89	0.90	0.79	0.042	481.7	All significant at 0.001, 2-tailed	For BDI 19: 0.14 Others: 0.27- 0.60. All covariance paths b/w factors: P <0.001
Higher order for 3-factor	2.28	0.94	0.92	0.87	0.89	0.79	0.045	514.1	All significant at 0.001, 2-tailed, except "concentration" (0.004)	BDI 19 = 0.14 Others: 0.26-0.59
Bifactor for 3-factor	2.10	0.95	0.93	0.89	0.91	0.73	0.042	479.4	For general factor, all P < 0.001; for other factors, P >0.05	For general factor: BDI 19 = 0.16 Others: 0.25-0.59
SA-C: Fig 2	2.16	0.94	0.93	0.88	0.89	0.80	0.043	492.7	All significant at 0.001, 2-tailed	BDI 19 = 0.14 Others: 0.27 -0.60
CA-S: Fig 3	2.3	0.94	0.92	0.88	0.89	0.79	0.045	510.4	All P <0.001, except 'concentration" (0.002)	BDI 19 = 0.16 Others: 0.30-0.60
Higher order for SA-C and CA-S(Fig 4)	2.28	0.94	0.92	0.87	0.89	0.79	0.045	514.1	All P < 0.001, except concentration (	0.14-0.59
Bifactor for SA-C	1.82	0.95	0.94	0.92	0.94	0.75	0.036	431.4	For general factor: P <0.001, except BDI 19 = 0.04. For other factors, mostly P > 0.05	General factor: BDI 19: 0.097 Others: 0.25-0.60
Bifactor for CA-S (Fig 5)	1.73	0.96	0.94	0.93	0.94	0.75	0.034	416.7	For general factor: P < 0.001, except BDI 19 = 0.02. For other factors, mostly P > 0.05	General factor: BDI 19: 0.14. Other items: 0.28-0.59
One-factor	2.28	0.94	0.92	0.87	0.89	0.79	0.045	514.1	All P < 0.001, except concentration (0.004)	BDI 19 = 0.14 Others: 0.28-0.59
Bahrain*	2.17	0.94	0.93	0.88	0.89	0.79	0.043	494.4	All P <0.001, except 'concentration" (0.003)	BDI 19 = 0.15 Others: 0.38-0.60. All covariance paths: P < 0.001
Dozois**	2.12	0.94	0.93	0.89	0.90	0.81	0.042	484.6	All P < 0.001, except 'concentration' (0.004)	BDI 19 = 0.14 Others = 0.27 -0.61 Covariance paths: P < 0.001

Notes: ^1^CMIN/DF = Chi-square divided degrees of freedom; ^2^GFI = "goodness-of-fit" index; ^3^AGFI = Adjusted GFI; ^4^TLI = Tucker -Lewis Index or Non-normed fit index; ^5^CFI = comparative fit index; ^6^PCFI = Parsimony adjusted comparative fit index; ^7^RMSEA = root mean square error of estimation; ^8^AIC = Akaike information criterion.

Standard values for the above fit indices are: GFI, AGFI, CFI, TLI: > 0.9

For others: PCFI > 0.6; CMIN/DF < 5; RMSEA < 0.08. In comparing models, the one with lesser AIC indicates better fit to the data.

* BDI items: 4,8,10-13,17 constituted the "overt emotional upset" domain

** BDI items 1-3, 5-9 and 13-14 loaded on the "C-A" factor; while items 4,10-12 and 15-21 loaded on the somatic-vegetative factor

The alpha coefficients of the two-factor models are as follows: (i) CA-S model: factor CA (No. of items = 16): 0.81; factor "S" (No. of items = 5): 0.49; (ii) SA-C model: factor "C" (No. of items = 9): 0.73, factor "SA" (No. of items = 12): 0.72.

The mean total BDI score was 15.5 (SD = 8.5), and median was 14. The mean scores for the items ranged from 0.26 to 1.1 (average 0.76) (Table 2). Using standard cut-off scores [12], 125 (20.0%) had moderate depression (score 21-30); 33 (5.3%) had severe depression (score 31-40), while 5(0.8%) had extreme depression (score 41-63). The BDI total score for women (16.2, SD = 8.8) was significantly higher than that for men (14.04, SD = 7.5) (t = 2.82, df = 622, P < 0.005). This significant gender trend was maintained for summary scores for the domains of the two-factor models (P < 0.01), except the cognitive factor of the SA-C model (P = 0.088).

**Table 2:** Psychometric characteristics of the BDI-II: N = 624

BDI-II item	Corrected item total correlation	Mean (SD)	% subjects scoring > 0
BDI 1: sadness	0.46	0.86(0.73)	70.0
BDI 2: pessimism	0.42	0.52(0.80)	36.4
BDI 3: past failure	0.48	0.49(0.76)	33.5
BDI 4: loss of pleasure	0.42	0.95(0.97)	62.0
BDI 5: guilty feelings	0.36	0.91(0.86)	60.4
BDI 6: punishment feelings	0.41	0.75(0.95)	45.7
BDI 7: self-dislike	0.51	0.40(0.73)	28.2
BDI 8: self-criticalness	0.36	1.1(0.87)	76.6
BDI 9: suicidal thoughts	0.45	0.26(0.54)	22.4
BDI 10: crying	0.39	0.73(1.1)	40.5
BDI 11: agitation	0.37	0.98(1.1)	63.5
BDI 12: loss of interest	0.39	0.64(0.79)	45.7
BDI 13: indecisiveness	0.52	0.98(0.89)	63.1
BDI 14: worthlessness	0.40	0.49(0.81)	29.3
BDI 15: loss of energy	0.50	0.99(0.81)	67.9
BDI 16: sleep pattern	0.43	0.83(0.82)	61.7
BDI 17: irritability	0.49	1.1(0.93)	72.3
BDI 18: appetite	0.37	0.68(0.84)	49.0
BDI 19: concentration	0.14	0.75(1.0)	44.6
BDI 20: tiredness	0.26	0.68(0.84)	49.0
BDI 21: loss of interest in sex	0.23	0.47(0.81)	30.8

All corrected item-total correlations were significant (P < 0.001); for items 1-18 (range of r: 0.36 -0.52) it was mostly 0.40 -0.49; it was lowest for "concentration" (0.14) (Table 2).

All correlations with the HSCL-25 domain scores were highly significant (r mostly > 0.5, P < 0.001) (Table 3). The summed scores of the cognitive factors of the two-factor models had significantly higher correlations with the depression score of the HSCL-25 (r: 0.66-0.70) than with the HSCL-25 anxiety score (r: 0.54-0.57) (Z = 3.9, P < 0.001).

**Table 3:** convergent validity: Pearson's correlations for domains of BDI-II with HSCL-25 anxiety and depression subscale scores: N = 624

BDI-II models	HSCL-25 anxiety subscale: r*	HSCL-25 depression subscale: r*	HSCL-25 total: r*
CA-S model: cognitive domain	0.57	0.70	0.69
CA-S model: somatic domain	0.48	0.57	0.54
SA -C model: cognitive domain	0.54	0.66	0.66
SA - C model somatic domain	0.56	0.65	0.66

* P < 0.0001

## Discussion

We analyzed the responses of 624 Arab college students to the BDI-II, in order to investigate whether the existing factor structures fit the data. We did this by comparing the "fit" of eleven models of depression at lower order (dimensional) and hierarchical levels to the data, using eight "fit" indices. We also examined the psychometric characteristics of the BDI-II. Our results were broadly in support of the majority findings in the literature, indicating that the multi-domain structure of the BDI-II is robust, the bifactor model is the best representation of the relationship between the items of depression, and the psychometric characteristics of the BDI-II are adequate. We note that, in exploratory factor analysis by principal axis factoring and oblique rotation for our data, four factors emerged, accounting for 41.8% of variance explained, and that these factors were effectively one-half of each of the two domains of the data for college students from the USA (data not shown) [12,16,17].

While the first - order factor dimensional models had mostly similar fit indices (AIC values: 481.7 -510.4), the original three - factor model had a slightly better fit. Although the higher - order version of these lower order models did not result in improved fit, the bifactor models did. Interestingly, the bifactor version of the CA-S model (derived from data of college students by Beck et al [12]) had the best fit indices, indicating the robustness of this model within samples of students across cultures. The loadings on the general factor of the bifactor model provide some insight into the nature of the specific domains of the BDI-II. First, we were surprised that for such a conservative culture, the item on sex (BDI-II 21) was apparently not much problematic for this age group [12,14], since it had highly significant loadings (regression weights on its lower order factor in the various models was 0.56 -0.89, P < 0.001) and the standardized regression weight on the general factor of the bifactor model was 0.27. However, along with the item on concentration and tiredness/fatigue, they constituted the lowest standardized regression weights (< 0.3), implying that they are poor indicators of the latent construct [56]. Second, the regression weights of the specific, uncorrelated factors of the BFM were not significantly different from zero, indicating that these lower order factors were very closely related to the general factor because the variance related to them was mostly explained by the general factor [47]. This supports the use of the total score for assessment of severity of depression [45,57]. However, the dimensional models from the lower order factors also had adequate fit to the data. The interpretation of these findings, according to the theory of bifactor models [46], is that, while the general factor of the bifactor model represents the common trait shared by all the items of the BDI (e.g., low positive affect - [58]), the lower order factors are independent sources of common variation (e.g., tendency to endorse cognitive or somatic symptoms) that reflect coherency among particular subgroups of symptoms. In line with this, Shafer[1] concluded from a meta-analysis of the factor structure of four popular depression rating scales, that these instruments can be conceptualized as measuring a single, higher order general depression factor, and at a lower level as measuring a number of specific depression symptom - factors. This pattern of relationship has been shown to be useful in settings, such as intelligence, externalizing disorders, health-related quality of life, and psychopathology [44,46,47,37,59,60]. Using the example of studies in attention deficit hyperactivity disorder [60], the clinical implication is that the symptom domains interact synergistically to give rise to the heterogeneous expression of clinical depression.

Finally, we have replicated the finding that the bifactor model tends to result in improved "fit" statistics in CFA [44,37,59]. In other words, the bifactor model appears to be emerging as the best representation of relationships in general constructs that are comprised of several highly related domains.

We have replicated the robust finding in the literature that the BDI-II is psychometrically sound across cultures, because the internal consistency was adequate, our mean item score was similar to the average for student samples, all corrected item -total correlations were significant (P < 0.001), there was adequate convergent/discriminant validity using the HSCL-25, and the women had significantly higher scores than the men [35].

The mean total BDI-II score for our subjects was much significantly higher than those of students reported from neighboring Iran (9.79, SD = 7.96, N = 125) [27], as well as those from North America, reported by Beck and colleagues [12] (12.56, SD = 9.93, N = 120), Dozois et al [18] (9.11, SD = 7.57), Whisman et al [16] (8.36, SD = 7.16, N = 576), and Storch et al [17] (11.03, SD = 8.17) (Effect sizes ranged from 0.34 to 0.91; 95% C.I. ranged from 0.14 to 1.03). While only one item was endorsed by over 50% of subjects in the Iranian report, eight items were endorsed by over 50% of our participants (Table 2). In the five-country European study of non-clinical samples, Nuevo et al [3] reported that the BDI-I mean scores ranged from 3.12(SD = 4.8; N = 1245) for Spain, to 8.51 (SD = 9.16; N = 456) for Ireland. Eight items were endorsed by 60.4%-70% of our subjects.

We have no specific explanation for the relatively high rate of depressive symptoms among our subjects. However, we note that in recent face-to-face interview-based reports on posttraumatic stress disorder (PTSD) among a representative sample of Kuwaiti military men, their wives and children, it was found that, six years after the First Gulf War, the prevalence of PTSD remained high among the subjects(31.5% for the men, 28% for their wives, and 14% anxiety/depression for their children) [61-63]. The speculation is that Kuwaitis may be prone to anxiety/depression because of their experience during the Iraqi occupation and the heightened security situation that persisted thereafter [61]. In a review of epidemiological studies of anxiety disorders in the Arab world, it was found that the prevalence of anxiety was highest in post conflict countries, such as Algeria, Palestine and Lebanon [64]. Furthermore, university students in two Arab countries (Lebanon and the UAE) had higher anxiety scores than comparison Canadian students [64].

### Limitations and strengths

Although our study was cross-sectional and based on only one population, our findings have merit because we performed the CFA in a standard manner, using a large sample size and with a broad variety of indices to judge the fitness of hierarchical and dimensional models to the data. However, our sample is different from the general population because it is made up of a homogenous group of individuals from one college. Hence, future studies in this setting should attempt to study other population groups in order to see how replicable the findings are in various population groups.

Although it has been noted that it is difficult to interpret what the general factor of the bifactor model measures [31], we suggest that the needed interpretation has been provided by theorists in the field, as indicated above [46,47,37,59], and that the success of the tripartite model of anxiety and depression [58,65] implies that low positive affect is a good proxy for the general factor.

## Conclusions

As alternative approaches for representing the multi-domain construct of depression, the broadly adequate fit of the various models shows that they have some merit. This implies that the relationship between the domains of depression probably contains hierarchical and dimensional elements. In support of this point, it has been reported that models are not mutually exclusive; they can coexist in different parts of the same complex model [47,66]. In line with this view, and using the example of externalizing disorders, Krueger and Piasecki [67] have suggested that a hierarchical spectrum model treats psychopathological variations as continuous and dimensional; and that the continuous variations are organized in a hierarchy. That is, while the general factor of the bifactor model represents the unifying, internalizing liability to depression, the specific factors represent the etiologic variables that undergird the phenotypic coherence of this liability[68]. The hierarchical model represented by the bifactor approach is emerging as the best way to account for the clinical heterogeneity of depression, and the adequacy of the psychometric characteristics of the BDI-II in our sample lends support to this view. This is in line with the emerging evidence that a hierarchical model is the best representation of affect and psychopathology [48,49,65,67].

## Competing interests

The authors declare that they have no competing interests.

## Authors' contributions

FAA conceived the study and supervised collection of data. FAA and JUO designed the study and analyzed the data. FAA and JUO drafted the manuscript. All authors read and approved the manuscript.

## Pre-publication history

The pre-publication history for this paper can be accessed here:

http://www.biomedcentral.com/1471-244X/10/60/prepub
